# Improved Empirical Formula Modeling Method Using Neuro-Space Mapping for Coupled Microstrip Lines

**DOI:** 10.3390/mi14081600

**Published:** 2023-08-14

**Authors:** Shuxia Yan, Fengqi Qian, Chenglin Li, Jian Wang, Xu Wang, Wenyuan Liu

**Affiliations:** 1 School of Electronics and Information Engineering, Tiangong University, Tianjin 300387, China; yanshuxia@tiangong.edu.cn (S.Y.); 2131070907@tiangong.edu.cn (C.L.); 2 School of Microelectronics, Tianjin University, Tianjin 300072, China; 3 Qingdao Institute for Ocean Technology, Tianjin University, Qingdao 266200, China; 4 School of Electronic Information and Artificial Intelligence, Shaanxi University of Science and Technology, Xi’an 710021, China

**Keywords:** mapping neural networks, modeling, optimization, coupled microstrip lines, microwave devices

## Abstract

In this paper, an improved empirical formula modeling method using neuro-space mapping (Neuro-SM) for coupled microstrip lines is proposed. Empirical formulas with correction values are used for the coarse model, avoiding a slow trial-and-error process. The proposed model uses mapping neural networks (MNNs), including both geometric variables and frequency variables to improve accuracy with fewer variables. Additionally, an advanced method incorporating simple sensitivity analysis expressions into the training process is proposed to accelerate the optimization process. The experimental results show that the proposed model with its simple structure and an effective training process can accurately reflect the performance of coupled microstrip lines. The proposed model is more compatible than models in existing simulation software.

## 1. Introduction

With the rapid development of electronic systems, circuit design requires high-performance microwave devices [1]. Most of the circuit designs are first simulated by simulation software to obtain the desired circuit performance and then physically fabricated [2]. Simulations not only make it possible to find the appropriate circuit parameters faster but also save labor and production costs in the physical manufacturing process [3]. Therefore, highly accurate and efficient microwave device models play a very important role in circuit design simulation [4,5,6,7]. Improving device modeling accuracy and shortening device design cycle have become major fields of research in microwave devices [8,9]. Traditional modeling methods consume a lot of human and computer resources by constantly trying and correcting to obtain an accurate model [10,11]. In addition, due to the lack of degrees of freedom, the models built by the traditional modeling methods often fail to meet the required accuracy. In order to satisfy the requirements of fast simulation and high model accuracy [12,13], advanced modeling methods need to be investigated. Coupled microstrip lines are widely used in microwave semiconductor devices for their small size, simple structure, stability and reliability [14,15]. However, the existing models of coupled microstrip lines for device design rely heavily on simulation software [16,17], which limits the flexibility of the models. This paper focuses on a new modeling method for coupled microstrip lines to efficiently build an accurate and highly compatible model.

Artificial intelligence and deep learning are now widely used in the field of microwave device modeling. Research on, development of, and innovation in intelligent approaches in microwave devices have become popular topics of research in this field [18,19,20]. Artificial neural networks (ANNs) are one of the early machine learning algorithms [21]. Modeling methods using ANNs are considered an effective alternative in the field of microwave device modeling [22,23]. ANNs have strong learning and generalization capabilities, and can learn the nonlinear relationship between the input and output of modeled microwave devices by optimizing weights. The trained model can accurately reflect the output responses of the modeled device. The more data that provide a nonlinear relationship within a certain range, the more accurate the prediction of the ANNs will be [24,25]. To avoid the large amount of data required for ANNs, Neuro-SM is proposed for modeling microwave devices [26,27,28]. Compared with traditional ANN modeling methods, the Neuro-SM method can effectively save training cost and improve the generalization capability of ANNs. Normally, accurate data obtained by the simulation or measurement of the modeled microwave devices are defined as the fine model, while the coarse model is expressed by empirical formulas that can match the fine model approximately. The Neuro-SM model consists of two parts: the coarse model and the MNNs. By adjusting the MNNs, the coarse model is gradually adapted to the characteristics of the fine model, enabling the Neuro-SM model to achieve both high accuracy and high simulation efficiency. This method has been widely used for device modeling in the microwave field [29,30,31,32,33,34].

The first presentation of the Neuro-SM modeling method is in [35], which modifies the modeled microwave device behavior with new space mapping formulas. If the performance of the coarse model is similar to the modeled microwave device, the Neuro-SM model matches the fine model well by introducing the input MNNs. The frequency MNNs added to the Neuro-SM model can enhance the frequency characteristics of the coarse model [36]. In [29], the coarse model introduces both the input and output MNNs in order to obtain a more accurate model. If there is a significant difference between the fine model and the empirical formulas of the coarse model, the existing Neuro-SM modeling methods cannot develop a precise model. More degrees of freedom of variables are required in input MNNs. Frequency MNNs can easily put the training process into an overlearning state. For output MNNs, it is difficult to achieve passivity in a passive device model. Therefore, novel modeling methods based on Neuro-SM are needed to cover the differences between the coarse and fine models.

This paper proposes an improved empirical formula modeling method for coupled microstrip lines. The correction values are added to the empirical formulas and the modified empirical formulas are used as the coarse model. The MNNs adjusts the correction values according to the input variables, improving their ability to learn and predict. In addition, an advanced training method is proposed to automatically optimize the weights of the MNNs in order to improve the efficiency of modeling. Modeling examples verify the effectiveness and feasibility of the improved empirical formula modeling method proposed in this paper.

## 2. Proposed Empirical Formula Modeling Method

**Figure 1:**
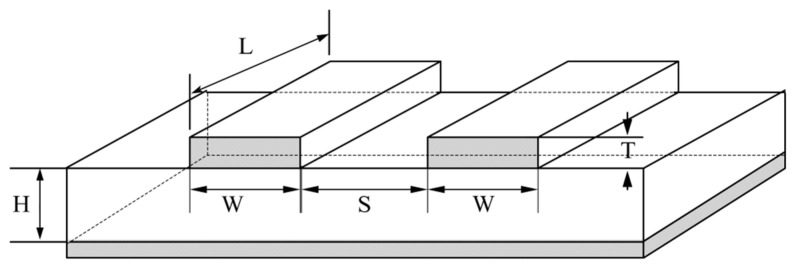
Physical structure diagram of the coupled microstrip lines [37].

The microstrip line is made on a dielectric substrate with height H and relative permittivity $\varepsilon_{r}$. A conductor strip of length L, width W, and thickness T is on one side, while a grounded metal plate is on the other side. The coupled microstrip lines consist of two parallel microstrip lines spaced S apart from each other. Let the normalized width be defined as u, i.e., u=W/H. Let the normalized gap be defined as g, i.e., g=S/H. Figure 1 shows the physical structure of the coupled microstrip lines.

The two microstrip lines in the coupled microstrip lines are close to each other, so there is a coupling phenomenon of electromagnetic signals when electromagnetic waves are transmitted. The coupled microstrip lines are surrounded by the nonuniform medium, so the transmitted electromagnetic waves are mixed modes with dispersion characteristics, which makes the analysis more complicated. For the convenience of analysis, the mode transmitted in the coupled microstrip lines is considered a transverse electric and magnetic field mode in this paper. It can be decomposed into odd-mode and even-mode modes when different excitation sources are applied to the coupled microstrip lines. In these operating states, the transmission between the two parallel lines is independent of each other and coupled with each other. These two transmission states are mathematically separated and studied in terms of symmetry and antisymmetry. The even-mode excitation means that the magnitude and phase of the incident source excitation at the symmetric port are the same, while the odd-mode excitation has the opposite magnitude and phase of the incident source excitation at the symmetric port. A mathematical method is used to analyze coupled microstrip lines, making the model independent of different simulation software and highly compatible.

### 2.1. Improved Empirical Formulas of the Coarse Model

The odd- and even-mode methods are used to develop the empirical formulas from geometric variables to generate responses. Some important empirical formulas are derived in this section. The effective permittivity for even-mode and odd-mode excitation is given by:

(1) $$\left\{\begin{array}{l} \varepsilon_{r}{}_{effe}=\left(\varepsilon_{r}+1\right)/2+\left(\varepsilon_{r}-1\right)/2*F_{e}\\ \varepsilon_{r}{}_{effo}=\left(\varepsilon_{r}+1\right)/2+\left(\varepsilon_{r}-1\right)/2*F_{o} \end{array}\right.$$

where $F_{e}$ and $F_{o}$ are parametric equations related to the geometrical variables, which are explained in [38].

Equations (2) and (3) represent the characteristic impedance and the characteristic capacitance for even-mode and odd-mode excitation, respectively:

(2) $$\left\{\begin{array}{l} Z_{Oe}=Z_{o}/\sqrt{\varepsilon_{r}{}_{effe}}/\left(1-Z_{o}*\phi_{e}/\eta_{o}\right)\\ Z_{Oo}=Z_{o}/\sqrt{\varepsilon_{r}{}_{effo}}/\left(1-Z_{o}*\phi_{o}/\eta_{o}\right) \end{array}\right.$$

(3) $$\left\{\begin{array}{l} C_{e}=\sqrt{\varepsilon_{r}{}_{effe}}/Z_{Oe}/c\\ C_{o}=\sqrt{\varepsilon_{r}{}_{effo}}/Z_{Oo}/c \end{array}\right.$$

where $\eta_{o}$ is defined as the wave impedance in vacuum, $Z_{o}$ expresses the impedance of the uniform microstrip line, c represents the speed of light, and $\phi_{e}$ and $\phi_{o}$ are parametric equations related to the u and g of the coupled microstrip line, which are explained in [38].

The substrate dielectric of the coupled microstrip lines remains unchanged, while the medium around the conductor strip of the coupled microstrip lines is completely replaced by air. In this case, the characteristic capacitance of even- and odd-mode excitation is given by:

(4) $$\left\{\begin{array}{l} C_{ae}=1/\left(Z_{Oe}*\sqrt{\varepsilon_{r}{}_{effe}}\right)/c\\ C_{ao}=1/\left(Z_{Oo}*\sqrt{\varepsilon_{r}{}_{effo}}\right)/c \end{array}\right.$$

The mutual inductance and the self-inductance are represented by Equation (5), and the mutual capacitance and the self-capacitance can be expressed by Equation (6):

(5) $$\left\{\begin{array}{l} L_{m}=\mu_{0}*\varepsilon_{0}/2*\left(1/C_{ae}-1/C_{ao}\right)\\ L_{0}=\mu_{0}*\varepsilon_{0}/2*\left(1/C_{ae}+1/C_{ao}\right) \end{array}\right.$$

(6) $$\left\{\begin{array}{l} C_{m}=1/2*\left(C_{o}-C_{e}\right)\\ C_{0}=1/2*\left(C_{o}+C_{e}\right) \end{array}\right.$$

where $\mu_{0}$ is defined as permeability of vacuum and $\varepsilon_{0}$ expresses the vacuum absolute permittivity.

The empirical formulas derived by the odd- and even-mode methods roughly match the fine model of the coupled microstrip lines, but when the operating frequency is too high or the input variables vary over a wide range, it takes a lot of time and computer resources to constantly try and correct the intermediate parameters in the empirical formulas. In most cases, the empirical formulas fail to build an accurate model. This method finds the intermediate parameters in the empirical formulas that have a large impact on the response of the coarse model through control variables. The correction values are considered as factors multiplied by the intermediate parameters in the corresponding positions of the empirical formulas, which improve the flexibility of the model. The improved coarse model consists of empirical formulas with correction values. The whole process of building the coarse model is performed in NeuroModelerPlus software. In the proposed method, correction values are added at the locations of relative permittivity, impedance, capacitance and inductance. For example, $L_{m}$ is a selected intermediate parameter, $\Delta L_{m}$ is the correction value, and the improved empirical formula is $L_{m}{}_{\_improved}=L_{m}*\Delta L_{m}$. By changing the values of the selected intermediate parameter, the response of the coarse model gradually approaches that of the fine model. The experimental result shows that the method gives enough degrees of freedom to the coarse model to make it more flexible to accurately match the fine model.

### 2.2. Improved Neuro-SM Model Structure

**Figure 2:**
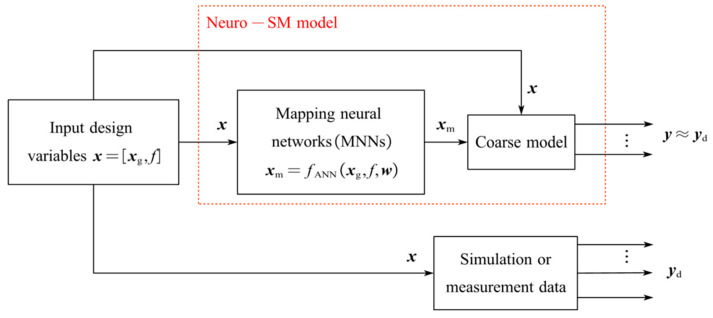
Schematic diagram of the improved Neuro-SM model structure.

To obtain an accurate model, the improved Neuro-SM model is proposed based on empirical formulas and correction values. Figure 2 is a schematic diagram of the improved Neuro-SM model structure. The parameter analysis method was used to determine the input variables that have significant effects on the response characteristics of the coupled microstrip lines. The input variables for the improved Neuro-SM model are defined as x, which includes the geometrical variables $x_{g}={\left[L,S\right]}^{T}$ and frequency variable f. x are the inputs to both the coarse model and the MNNs. The vector $x_{m}$ represents the correction values added to the empirical formulas. The output variables y are the responses of the improved Neuro-SM model.

MNNs with a simple structure can accurately represent the nonlinear relationship between x and $x_{m}$. The MNNs adjust the internal weights w according to the different x, and then change $x_{m}$. The adjusted $x_{m}$ and x are fed into the coarse model, and y are finally generated. Through the training process of the MNNs, the outputs y of the coarse model match the outputs $y_{d}$ of the fine model well. The nonlinear relationship between x and $x_{m}$, which is adjusted by the MNNs, is represented by $f_{ANN}$. The expression is shown as:

(7) $$x_{m}=f_{ANN}(x,w)$$

where w denotes the internal weights of the MNNs and $f_{ANN}$ denotes a multilayer perceptron neural network that uses the sigmoid function as an excitation function to arbitrarily approximate the nonlinear relationship between input and output [39].

### 2.3. The Proposed Training Method

In the proposed method, the most critical step in modeling is to obtain the internal weights of the MNNs so that the outputs of the developed model are constantly close to those of the desired device. The training process determines not only the learning effect of the model but also the prediction effect. In this paper, the evaluation criteria for model learning and prediction ability are usually presented in term of training error $E_{Tr}$ and test error $E_{Te}$, and can be formulated as:

(8) $$E\left(w\right)={\left[\frac{1}{N_{s}N_{y}}\sum_{n=1}^{N_{s}}{\sum_{k=1}^{N_{y}}\left|\frac{Y_{n}^{k}(w)-Y_{nD}^{k}}{{\left|Y_{D}^{k}\right|}_{\max}}\right|}^{2}\right]}^{\frac{1}{2}}$$

where $Y_{n}^{k}(.)$ and $Y_{nD}^{k}$ are the responses of the improved Neuro-SM model and the responses of the fine model, respectively. ${\left|Y_{D}^{k}\right|}_{\max}$ represents the maximum value of the absolute value of the fine model responses. The superscript k is the index of the output response, and $N_{y}$ is the total amount of output response. The subscript n indicates the index of the modeled data $n=1,2,\ldots \ldots ,N_{s}$, where $N_{s}$ is the total amount of modeled data. During the optimization process, the internal weights of the MNNs are continuously adjusted by using different optimization algorithms to reduce the error until the error meets the accuracy requirements.

To speed up the training process, the internal weights w of the MNNs are treated as optimization variables. The first-order derivative ∂y/∂w is used to speed up the search for the optimal variables. Since the input variables for the fine and coarse models are the same, the subscript c is added to the signs of the input variable of the coarse model in Equation (9). The first-order derivative of the fine model outputs $y_{d}$ with respect to the optimization variables w is given by:

(9) $$\frac{\partial y_{d}{}^{T}(x_{g},f,w)}{\partial w}=\frac{\partial y_{d}{}^{T}(y)}{\partial y}(\frac{\partial y_{}^{T}(x_{gc},f_{c})}{\partial x_{gc}}\frac{\partial x_{gc}^{T}(x_{g},f,w)}{\partial w}+\frac{\partial y_{}^{T}(x_{gc},f_{c})}{\partial f_{c}}\frac{\partial f_{c}(x_{g},f,w)}{\partial w})$$

where $\partial y_{d}{}^{T}(y)/\partial y$ is the derivative of the fine model outputs $y_{d}$ with respect to the coarse model outputs y. $\partial y_{}^{T}(x_{gc},f_{c})/\partial x_{gc}$ and $\partial y_{}^{T}(x_{gc},f_{c})/\partial f_{c}$, respectively, represent the derivative of the coarse model outputs y with respect to the input variables of the coarse model $x_{gc}$ and the frequency variable of the coarse model $f_{c}$. $\partial x_{gc}^{T}(x_{g},f,w)/\partial w$, and $\partial f_{c}(x_{g},f,w)/\partial w$ represents the derivative of the input variables of the coarse model $x_{gc}$ and the frequency variable of the coarse model $f_{c}$ with respect to the optimization variables w, respectively.

### 2.4. The Whole Process of the Proposed Modeling Method

**Figure 3:**
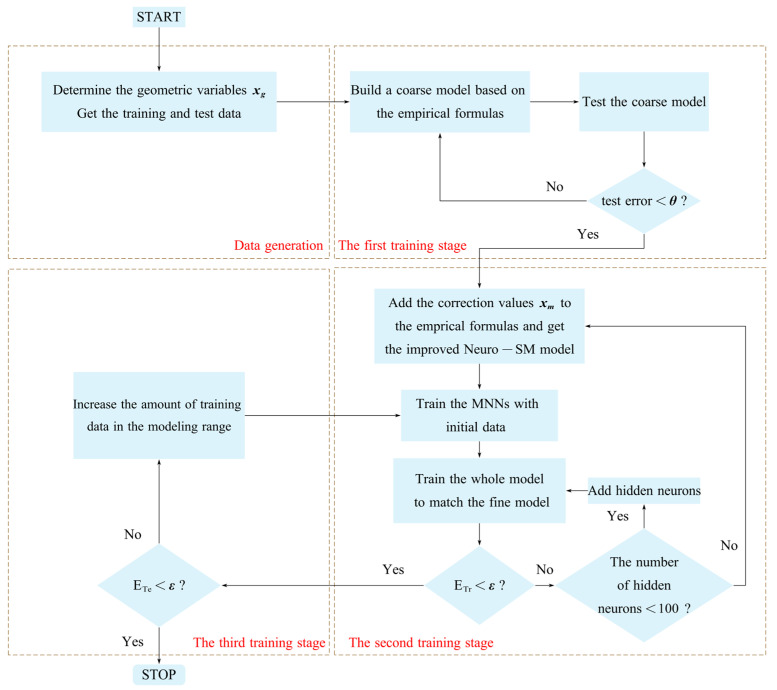
Flowchart of the whole process of the proposed modeling method.

The flowchart of the whole process of the proposed modeling method is shown in Figure 3. In the data generation section, the geometric variables that have significant effects on the responses of the coupled microstrip lines are first determined. The training and test data for the proposed model are generated using the design of experiments (DOE) method [40]. The DOE method can generate the geometric parameters of an orthogonal distribution, which ensures that the training data can represent the entire modeling range approximately. The proposed Neuro-SM can learn the nonlinear relationship between x and $y_{d}$ with the training data, and the predictive capability of the model is verified using the test data. Test data are within the training data range and different from the training data.

The first step of the training process is to build a coarse model according to the empirical formulas. The odd- and even-mode methods derive all empirical formulas used for modeling, as described in Section 2.1. If the test error of the initial coarse model is higher than a user-defined threshold θ, it returns to the derivation part of the empirical formulas. Otherwise, the development of the coarse model has been preliminarily completed.

The second step is to complete the construction and train the improved Neuro-SM model in the training process. Based on the improved empirical formulas for the coarse model in this paper, the correction values are added at appropriate locations. After that, the whole construction of the model is completed according to the structure of the improved Neuro-SM model. Before the whole model is trained, unit MNNs are first developed. The test error of the Neuro-SM model with unit MNNs is identical to that of the coarse model. The input data of the MNNs are x and the outputs are the value 1. The number of the MNN outputs is the same as the number of $x_{m}$. The initial data are randomly generated in MATLAB software, which has a larger range than the training data. The training data are used to adjust the internal weights of the MNNs, which affect the intermediate parameters with the correction values, so that the responses of the coarse model are consistent with the fine model. When $E_{Tr}$ does not satisfy the user-defined threshold ε while the number of hidden neurons is fewer than 100, the number of hidden neurons can be increased to increase the nonlinear degree of the proposed model. If the number of neurons has reached 100, the process returns to increase the amount of correction values and retrains the new model. The training process will not stop until $E_{Tr}$ meets ε. This process focuses on finding the number of correction values and hidden neurons that minimize the training error of the proposed model. The effectiveness of the proposed model is demonstrated by obtaining the best results with the fewest correction values and hidden neurons.

In the third training stage, the model is tested against the test data to verify that it makes good predictions for untrained data in the modeling range. If $E_{Te}$ satisfies ε, the model development is complete. If $E_{Te}$ does not satisfy ε, it means that the training data are insufficient and the amount of training data needs to be increased for retraining. The smaller the test error, the better the generalization ability of the proposed model.

## 3. Experimental Verification

In this section, the experimental verification is performed by modeling the coupled microstrip lines. The fine model is the coupled microstrip line structure built in Advance Design System (ADS) simulation software and the coarse model is the model with empirical formulas and correction values. In this experiment, the line length L and coupled lines spacing S are used as geometric variables, while f is a frequency variable. In this paper, the DOE method is used to generate training and test data ranges for geometric and frequency variables, respectively. The data ranges are shown in Table 1. The training and test data used for the proposed model are obtained by simulation in ADS software. To ensure that the test data are untrained, different starting and ending points are chosen for the training and test data with the same intervals.

During the training process, finding a suitable set of weights can effectively reduce the difference between the coarse model and the fine model. Therefore, it is essential to choose a suitable number of correction values. The choice of correction values depends on the influence of the intermediate parameters of the empirical formulas on the **S**-parameter results. The correction values as factors should be multiplied by the intermediate parameters, which significantly affect the **S**-parameters. When correction values are added to intermediate parameters that have less impact on the responses, it not only wastes computer resources but also results in slower modeling.

Table 2 shows the training and test errors for different numbers of correction values and hidden neurons for the proposed model. When the number of correction values is fixed, the number of hidden neurons is continuously varied to find the minimum number of hidden neurons, making the training error and test error satisfy the user-defined threshold. The training and test errors are compared at different correction values to find the most appropriate number of correction values. According to Table 2, we can find that the result with only 15 hidden neurons when the correction value is 12 is much better than the result with 35 hidden neurons when there are 8 correction values. When the number of correction values is increased to 15, the result with 18 hidden neurons is not as good as the result with 12 correction values. The result shows that too few correction values cannot satisfy the nonlinear relationship of the coupled microstrip lines, while too many correction values represent high nonlinearity and complex structure of the MNNs, leading to lower accuracy.

At a fixed number of correction values of 12, the errors for different numbers of hidden neurons are shown in Table 3. When the number of hidden neurons is 10, the training and test errors of the model are higher than that of the model with 15 hidden neurons. This indicates that the nonlinear relationship between the input and output of the proposed model cannot be accurately expressed when the number of hidden neurons is small. However, when the number of hidden neurons is increased to 20 or 25, the training errors decrease while testing errors increase significantly. Because the models in this case are in the overlearning state, fewer hidden neurons are needed to retrain the model. It can be concluded that the best result of the proposed model can be achieved when the number of correction values is 12 and the number of hidden neurons is 15.

The correction values at the three frequency points for the geometric variables $x_{g}={\left[10.8,0.022\right]}^{T}(\mathrm{mm})$ are shown in Table 4. The results show that the correction values change with the frequency, making the coarse model responses at each frequency point fit the fine model responses well. Numerically, the correction values vary around 1, which proves that the choice of the correction values is appropriate. Small changes in the correction values can lead to large changes in response, thus ensuring the smoothness of the model output.

The feasibility of the proposed model is compared using two existing modeling approaches based on Neuro-SM. Model 1 adds the MNNs to the coarse model input, while model 2 adds the MNNs to both the coarse model input and output [32,38]. The proposed model achieves the lowest training and test error, as shown in Table 5. Comparing the results of 15 hidden neurons and 55 hidden neurons in model 1, it is found that increasing the number of hidden neurons did not reduce the training error, but significantly increased the test error. Model 1 is in the overlearning state. The error comparison of model 2 reveals that the accuracy of the model is not significantly improved only by increasing the number of hidden neurons in the input MNNs. By increasing the number of hidden neurons in the output MNNs, the training error of model 2 is reduced, but the test error fails to meet the requirement. This indicates that the amount of data used to train the model is not enough. Additional modules are needed to ensure model passivity due to the introduction of output MNNs. The above conclusions demonstrate that the proposed modeling method can accurately match the fine model while keeping the device passive.

**Figure 4:**
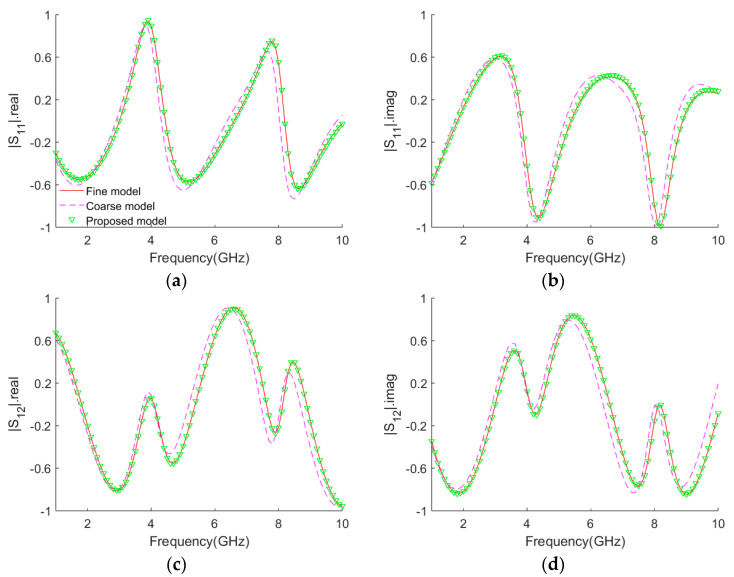
Comparisons of **S**-parameters among the fine model, the coarse model and the proposed model at $x_{g}={\left[10.83,0.022\right]}^{T}(\mathrm{mm})$. (**a**) $\left|S_{11}\right|\cdot real.$ (**b**) $\left|S_{11}\right|\cdot imag.$ (**c**) $\left|S_{12}\right|\cdot real.$ (**d**) $\left|S_{12}\right|\cdot imag$.

The comparison of the **S**-parameter responses among the fine model, the coarse model and the proposed model for the test data $x_{g}={\left[10.83,0.022\right]}^{T}(\mathrm{mm})$ is shown in Figure 4. The fine model is shown as a red line, the coarse model as a magenta dashed line and the proposed model as a green down triangle. It can be seen that there is a certain gap between the coarse model and the fine model, while the **S**-parameter responses of the proposed model and fine model are basically consistent. The matching results with the test data in the modeling range illustrate the feasibility of the improved empirical formula modeling method for coupled microstrip lines.

To verify the efficiency of the modeling method, computation time comparisons between the fine model in High-Frequency Structure Simulator (HFSS) software and the model built with the proposed method are shown in Table 6. The proposed model is developed with 25 sets of EM data generated by the DOE method, and the modeling time is 19.7 m. The trained proposed model can be used instead of the EM model in circuit design, because the two models have the same characteristics in the modeling range. From Table 6, it can be seen that the proposed model consumes less time than the fine model in the HFSS software when generating the same data. The more data that are needed, the more obvious the advantage of the proposed model. The proposed model applied into circuit design can significantly reduce the simulation time and thus shorten the device design cycle.

## 4. Conclusions

In this paper, an efficient modeling method based on empirical formulas is proposed. Correction values are added to the empirical formulas, increasing the flexibility of the coarse model. The improved Neuro-SM model establishes a nonlinear relationship between the coarse and fine models through the input MNNs, avoiding a slow trial-and-error process. Analytical expressions are used for training methods and sensitivity analysis, and the internal weights of the MNNs are optimized effectively. The proposed model uses fewer variables and provides more accurate results than existing Neuro-SM modeling methods. The coupled microstrip line model obtained by the proposed method is independent of any simulation software and has high compatibility, which expands the application of the model. In practice, this proposed modeling method can be used for other microstrip lines or microwave devices where equivalent circuits or empirical formulas exist, and accurate and efficient models can be obtained with the proposed training process.

## Figures and Tables

**Table 1:** Ranges of training and test data for the coupled microstrip line modeling.

Variable Type	Data Type	L (mm)	S (mm)	f (GHz)
Input variables	Training data	10:1.67:20	0.0178:0.0089:0.0711	1:0.1:10
	Test data	10.8:1.67:17.5	0.022:0.0089:0.057	

**Table 2:** Training and test errors for different numbers of correction values and hidden neurons.

Number of Correction Values	Number of Hidden Neurons	Training Error	Test Error
8	35	1.02%	1.25%
10	25	0.85%	0.91%
12	15	0.54%	0.55%
15	18	0.61%	0.68%

**Table 3:** Errors for different numbers of hidden neurons when the correction values are fixed at 12.

Number of Hidden Neurons	Training Error	Test Error
10	1.89%	1.92%
15	0.54%	0.55%
20	0.51%	1.90%
25	0.45%	2.31%

**Table 4:** The correction values at the three frequency points.

Correction Values	f=1 GHz	f=5 GHz	f=10 GHz
$\Delta \varepsilon_{r}{}_{effe}$	1.00453	1.00712	1.00686
$\Delta \varepsilon_{r}{}_{effo}$	0.987161	0.989964	1.00512
$\Delta Z_{Oe}$	1.00457	1.01083	1.03535
$\Delta Z_{Oo}$	1.01008	0.998378	0.950367
$\Delta C_{e}$	1.00453	1.00729	1.00744
$\Delta C_{o}$	0.987346	0.990366	1.00582
$\Delta C_{ae}$	1.0053	0.994982	0.966435
$\Delta C_{ao}$	1.01865	1.02539	1.05195
$\Delta L_{0}$	1.00796	1.00766	1.00403
$\Delta L_{m}$	1.00555	1.00739	1.00805
$\Delta C_{0}$	0.97079	0.975827	0.984218
$\Delta C_{m}$	1.05755	1.05757	1.06318

**Table 5:** Comparison of the training and test errors for 4 models.

Model Type	Hidden Neurons in Input Mapping	Hidden Neurons in Output Mapping	Training Error	Test Error
Coarse model	—	—	19.37%	18.32%
Existing model 1	15	—	6.71%	6.65%
	55	—	5.98%	10.22%
Existing model 2	15	15	2.85%	2.51%
	55	15	2.09%	1.86%
	15	55	0.58%	1.04%
Proposed model	15	—	0.54%	0.55%

**Table 6:** Comparison of computation time between the model in HFSS and the proposed model.

Sets of Data	Computation Time	
	Model in HFSS	Proposed Model
1	0.8 m	19.7 m + 0.02 s
50	35.05 m	19.7 m + 0.11 s
100	77.08 m	19.7 m + 0.22 s
